# Continuous sleep tracking in digital CBT-I: Efficacy and insights from a naturalistic-environment study

**DOI:** 10.1016/j.ijchp.2025.100646

**Published:** 2025-11-06

**Authors:** Alexandra Hinterberger, Esther-Sevil Eigl, Aniko Szeko, Pavlos I Topalidis, Manuel Schabus

**Affiliations:** a Laboratory for Sleep, Cognition & Consciousness Research, Department of Psychology, Paris Lodron University of Salzburg, Austria; b Department of Psychology, Sigmund Freud University Vienna, Austria; c Centre for Cognitive Neuroscience Salzburg (CCNS), University of Salzburg, Austria

**Keywords:** Insomnia, CBT-I, Digital program, Mhealth, Sleep tracking

## Abstract

**Background:**

Insomnia is highly prevalent, yet few receive cognitive behavioral therapy for insomnia (CBT-I) due to limited treatment availability. Unguided digital CBT-I offers an accessible alternative to traditional face-to-face therapy. Research in this area often relies on either subjective sleep measurements (e.g., sleep diaries) or controlled single-night lab studies. This study examines the effectiveness of a novel app-based CBT-I program combining therapy with continuous subjective and objective sleep tracking via a heart rate (HR) sensor in a naturalistic setting.

**Methods:**

Eighty-eight participants (56.8 % female) aged 20–85 years (M = 49.9 ± 13.10) completed an 8-week app-based CBT-I intervention with continuous sleep tracking (sleep diaries and HR sensor), followed by a 2-week follow-up. Assessments at baseline, post-intervention, and follow-up included sleep related problems, psychological strain, quality of life, and dysfunctional beliefs.

**Results:**

Insomnia prevalence dropped from 92 % at pre-intervention to 67 % at follow-up. Improvements were observed in subjective sleep quality (p < .001, r = 0.59), dysfunctional beliefs (p < .001, r = 0.48), quality of life (p’s < .002, r’s > 0.33), psychological strain (p < .001, r = 0.43), depression (p = .010, r = 0.27), and anxiety (p = .003, r = 0.32). While sleep diary data showed improvements in various sleep parameters, objective data revealed statistical trends towards a reduced total sleep time (TST; p = .083, r = 0.19), driven by sleep restriction, and light sleep (p = .089, r = 0.18). Using continuous sleep monitoring we additionally found relevant changes during the intervention levels for subjective wake after sleep onset, sleep efficiency as well as objective TST.

**Conclusion:**

Findings support the effectiveness of app-based CBT-I and suggest that continuous objective sleep tracking over weeks can reveal previously undetected sleep and well-being improvements in real-world settings.

## Introduction

Sleep problems in general and insomnia in particular pose an enormous burden on the individual and on society. With around 10 % of people qualifying for a clinical diagnosis of an insomnia syndrome according to diagnostical guidelines (i.e., experiencing difficulties initiating and/or maintaining sleep and/or early awakenings at least 3 times a week for at least three months, accompanied by daytime impairments such as fatigue, memory and concentration issues, emotional irritability), insomnia is considered the most common sleep disorder among western countries, with higher prevalence in women and middle to higher age (Riemann et al., 2023). Additionally, up to 20 % of the population suffers from symptoms of insomnia without necessarily meeting criteria for a medical diagnosis (Morin & Jarrin, 2022). Furthermore, the prevalence of sleep problems like insomnia has dramatically increased over the past decades (Dopheide, 2020).

Insomnia is associated with increased physical health issues, such as an increased risk for cardiovascular diseases (Sofi et al., 2014), but also mental health problems; insomnia patients have a 3-fold risk of developing depression and anxiety (Hertenstein et al., 2019). As affective disorders and insomnia are mutually dependent, patients may potentially experience a vicious cycle because of which both disorders may become chronic.

Furthermore, insomnia causes high societal and economic costs, including direct costs such as medical treatment costs as well as indirect costs due to increases in sick leave, work accidents, reduced productivity or absenteeism. People affected by insomnia lead to up to 12-fold costs compared to healthy sleepers, resulting in annual costs of insomnia of e.g., up to €60 billion in the US (Kessler et al., 2011).

Various options are available for the treatment of insomnia, including pharmacological (e.g., benzodiazepines, z-substances) and non-pharmacological options (e.g., psychotherapy). Yet, current guidelines do not recommend pharmacological treatment for >4 weeks or 3 months, depending on the substance, but rather define psychotherapy, i.e., cognitive behavioural therapy for insomnia (CBT-I) as the first line treatment for chronic insomnia (Riemann et al., 2023). CBT-I thereby combines core elements such as psychoeducation/sleep hygiene, relaxation exercises, cognitive training to address dysfunctional thoughts about sleep, as well as behavioural elements such as stimulus control and sleep restriction in order to restructure sleep habits.

Delivered in face-to-face (F2F) individual or group settings CBT-I has been shown to be highly effective in improving scores of the Insomnia Severity Index (ISI) and Pittsburgh Sleep Quality Index (PSQI), and sleep parameters such as total sleep time (TST), sleep onset latency (SOL), wake after sleep onset (WASO), sleep efficiency (SE) (Okajima et al., 2011; van Straten et al., 2018), as well as deep sleep percentage and duration (Cervena et al., 2004). Both subjective (i.e., sleep diary-derived) and objective (i.e., polysomnography-(PSG)/actigraphy-derived) sleep parameters seem to be susceptible to change and improvement, although subjective variables usually show faster and much greater improvements (Mitchell et al., 2019). Besides sleep-related parameters, CBT-I has furthermore been shown to alleviate symptoms of depression and anxiety – affective disorders that are highly comorbid with insomnia (Hertenstein et al., 2022).

However, unfortunately not many of those affected by insomnia get referred to CBT-I. One major problem is, that only roughly 30 % of people suffering from sleep problems seek help from their family doctor or general practitioner (GP), with only 15 % seeing a doctor to talk about insomnia complaints in particular (Marschall et al., 2017). Most people seeking medical help receive pharmacological treatment (50 %), and only 38 % are advised to CBT-I. The lack of trained clinicians able to perform 2F2 CBT-I or long geographical distances further pose a problem to providing adequate non-pharmacological care for people affected by insomnia (Dopheide, 2020).

In order to close this gap in care, validated digital CBT-I (dCBT-I) programmes, i.e., offering sleep therapy via a smartphone application (e.g., (Espie et al., 2012; Hinterberger et al., 2023; Lorenz et al., 2019) or website (e.g., (Eigl et al., 2023)), represent a low-threshold and important extension to F2F CBT-I, especially regarding the stepped care model of insomnia treatment distribution in order to prevent chronification of insomnia disorder (Espie, 2009). In fact, current guidelines define both F2F CBT-I as well as digitally administered CBT-I, i.e. dCBT-I, as firstline treatment; meaning the mode of administration of CBT-I is of secondary importance as long as CBT-I is the first treatment of choice (Riemann et al., 2023). Several meta-analyses investigated the efficacy of dCBT-I on insomnia severity, and sleep parameters such as SE, SOL, and WASO (e.g., Soh et al., 2020; Zachariae et al., 2016) and usually find similar effects sizes as compared to F2F CBT-I (Luik et al., 2017; Simon et al., 2023).

It is, however, to be noted, that investigated sleep parameters are usually subjective, i.e., derived with a sleep diary, and therefore do not represent sleep in an objectively measured manner, which can significantly differ from the personal experience, especially in people suffering from insomnia. It has, for example, been shown, that insomnia patients tend to overestimate their SOL, and furthermore underestimate their TST, when compared to objective sleep measurement (Harvey & Tang, 2012). In order to get a full picture of a person’s sleep pattern, it is therefore necessary to assess both, the subjective as well as the objective side of sleep.

Traditional approaches to dCBT-I, however, usually only include a sleep diary as a form of subjective sleep measurement in digital programs for the treatment of insomnia. The integration of continuous objective sleep measurement therefore represents a novel approach to digital insomnia therapy, that is yet to be systematically evaluated, however, presents the unique opportunity to objectively monitor sleep throughout different therapy phases. In recent years, wearable technology has increasingly been integrated into digital interventions for insomnia, particularly digital CBT-I. Several studies have already employed wearables as part of their study protocols (e.g., Ito-Masui et al. (2023); Kang et al. (2017); Luik et al. (2018)). However, in most cases, wearable devices are primarily used as objective measurement tools to complement subjective sleep diaries or pre- to post-intervention assessments, rather than as active components of the therapeutic process. The potential of wearables to provide individualized, real-time feedback and thereby enhance engagement and adherence has so far received limited empirical attention. Integrating wearable-derived sleep data into digital CBT-I programs in an interactive, feedback-oriented manner may therefore represent a valuable next step toward more adaptive and personalized sleep interventions.

Digital CBT-I programs can be guided or unguided, meaning they may include assistance of e.g., a psychologist, psychotherapist, medical worker through chatting/text messaging, (video) calls or other forms of digital but personal contact. This personal contact may enhance adherence; however, it still involves human resources, potentially making it more difficult to cover the growing number of insomnia patients. In contrast, unguided dCBT-I programs are designed to be fully automated and require little to no interaction with trained clinicians, facilitating low-threshold access to necessary treatment, as such programs can be used regardless of time and place. According to a recent meta-analysis comparing different modalities of CBT-I both guided and unguided digital CBT-I programs yielded medium effect sizes regarding improvements in insomnia severity (Simon et al., 2023), indicating that both modalities are effective in improving symptoms of insomnia. Regarding its efficacy, digital CBT-I therefore represents a low-threshold addition to the well-established F2F CBT-I.

Digital CBT-I programs have been broadly studied over the past years, oftentimes in very controlled and standardized settings, meaning e.g., strict inclusion and exclusion criteria, such as the exclusion of (severe) comorbid (sleep) disorders or exclusion of people who do not qualify for an insomnia diagnosis, people taking medication, drinking or smoking (e.g., (Espie et al., 2012; Koffel et al., 2018; Ritterband et al., 2017)). While this approach allows for a clean investigation and therefore better interpretation of effects, it is unfortunately far from the real world and does not necessarily represent the general population. It is therefore important to also investigate the efficacy of digital CBT-I programs in more naturalistic settings, which includes people with varying degrees of sleep problems, comorbid (sleep) problems, different ages, different motivations, as well as different sleeping patterns, such as bedpartners, children or pets in bed, sleep medication, habitual consumption of alcohol and/or other psychoactive substances, etc., as people reaching for low-threshold digital programs will most probably not be able to complete their therapy in controlled environments and in order to potentially learn about effects that might be missed in highly standardized settings.

The objective of the present paper therefore is 1) to investigate the efficacy of a novel unguided digital program for the treatment of insomnia including continuous subjective as well as objective sleep monitoring 2) in a naturalistic, real-world setting at home.

## Methods

### Participants

**Fig. 1:**
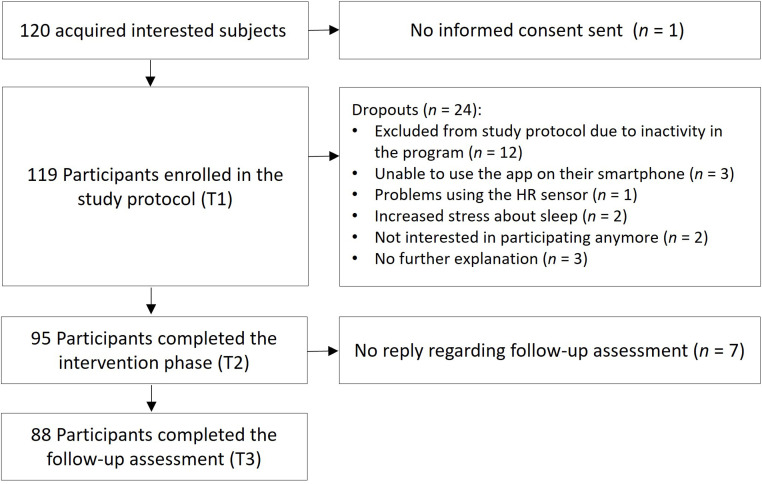
Flow of participants.

A total of 120 people were recruited through public media and University announcements, out of which 119 entered the study protocol. Subjects were included in the study, when the following criteria were met: Being at least 18 years of age and owning a smartphone (for downloading and using the study app). Sleep complaints were not defined as an explicit requirement for participation in this unguided field study in a naturalistic environment in order to mimic the real-world interest and engagement of users with the program regardless of a clinical cut-off. In practice, individuals engaging with digital CBT-I programs such as the one investigated here will not all suffer from severe sleep disturbances or meet diagnostic criteria for an insomnia disorder. However, most of the sample showed at least subthreshold levels of insomnia (see below), which was expected given the self-selection bias inherent in studies on digital sleep interventions. 88 participants completed all 3 main testing points over the course of 10 weeks and were therefore included in the final sample, resulting in a dropout rate of roughly 27 %, which is in line with other studies investigating digital CBT-I reporting a dropout of e.g., 44 % (Simon et al., 2025) or 24 % (Kater et al., 2025). For the detailed flow of participants, please see Fig. 1.

The sample consisted of 50 females (56.8 %) and 38 males (43.2 %), with an age range of 20–85 years (M = 49.9, SD = 13.10). Most participants had a university degree (n = 56; 63.6 %); the sample can therefore be considered highly educated. Almost half of the sample (n = 42; 47.7 %) was taking medication, some of which had prescribed psychoactive (n = 16; 18 %), or prescribed sleep medication (n = 6; 16.8 %).

Regarding baseline characteristics, according to the PSQI (Buysse et al., 1989) 44.3 % (n = 39) of participants reported poor sleep quality (PSQI: 6–9), and 38.6 % (n = 34) qualified as chronic poor sleeper (PSQI ≥ 10), while 17.1 % (n = 15) reported good sleep quality. Consistent with the ISI (Dieck et al., 2018) 92 % of the sample experienced various degrees of insomnia: 10.2 % (n = 9) suffering from severe clinical insomnia (ISI ≥ 22), 42 % (n = 37) experiencing moderate insomnia (ISI: 15–21), and 39.8 % (n = 35) experiencing subthreshold insomnia (ISI: 8–14), while 8 % (n = 7) did not have clinically relevant insomnia complaints (ISI: ≤ 7).

The study was conducted according to the Declaration of Helsinki and approved the Ethical Committee of the University of Salzburg. All participants gave informed consent prior to being included in the study protocol. The sample size fulfilled the statistical power requirements for middle and strong effects ( Kang, 2021).

### Study design and procedure

**Fig. 2:**
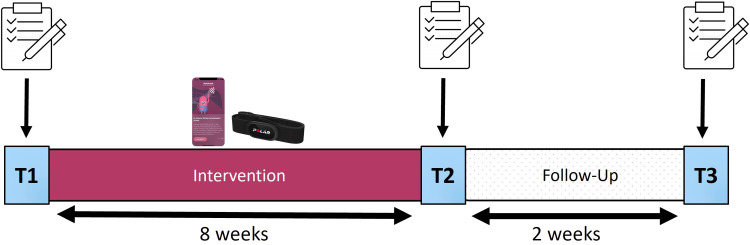
Study Design Notes: The 10-week study protocol was divided into an 8-week intervention phase and a 2-week follow-up period by three main testing time points: T1 marks the beginning of the study and therefore the intervention phase, T2 marks the end of the intervention phase, and T3 marks the follow-up assessment and the end of the study. For each of the main testing points a group of online questionnaires was conducted. During the 8-week intervention phase, participants used the app-program including continuous sleep monitoring using a heart rate sensor. At T2 access to the app-program was blocked in order to ensure reliable follow-up measurements.

In order to investigate the efficacy of a smartphone app including an unguided digital CBT-I program as well as continuous sleep measurement in the field, we conducted a simple study protocol from February 2023 to June 2023, consisting of an 8-week intervention phase, followed by a 2-week follow up period, and divided into three main testing points: Pre-intervention (T1), post-intervention (T2) and a final assessment after the follow-up period (T3). At each of the main testing points a group of online questionnaires was completed by the participants (more information: see Fig. 2). In this naturalistic setting we did not aim for an additional control group due to compliance problems since users would have to conduct daily sleep measurements over the course of 8 weeks without available feedback and benefit to them.

Throughout the intervention phase, participants engaged with the app’s dCBT-I program and applied a heart rate sensor (Polar® H10, Polar Electro; Kempele, Finland) for daily objective sleep monitoring. A concise sleep diary was filled in every morning, capturing subjective sleep quality and sleep metrics. Post-intervention, app access was blocked, so participants could only use the app between T1 and T2, to ensure valid follow-up measures.

The smartphone app’s (NUKKUAA, now: sleep²; NUKKUAA GmbH, Austria) sleep intervention program is tailored for addressing sleep complaints such as insomnia. The program is structured in consecutive levels, each offering short daily exercises based on core elements of CBT-I. This includes videos and short blogs on psychoeducation and sleep hygiene on topics such as how sleep changes across the lifespan or what hinders good sleep, audio relaxation techniques e.g., breathing exercises or progressive muscle relaxation, chat-bots providing cognitive training covering e.g., cognitive strategies to manage or reframe circling thoughts or introducing sleep restriction, sleep recommendations focusing on sleep hygiene and stimulus control, as well as short self-reflexion exercises at the end of each level. Every level presents one video, one auditory relaxation exercise, one chat-bot, two short blog posts covering topics like chronotypes, sleep myths or the comorbid symptoms of depression or anxiety, one sleep recommendation, such as keeping a regular sleep-wake cycle or only using the bed for sleeping, and one short exercise to reflect on the progress made within the current level. Within one level, users are free to choose the order or time of completing the exercises, in order to enable flexible engagement with the sleep training. Only the self-reflexion exercise was accessible after completing all other exercises of the current level. The primary program comprises six levels. Since one level can be completed within one week when engaging daily with the app the program was designed for completion within a minimum of 6 weeks. Higher levels (level 7 and higher), could theoretically be reached by participants when engaging daily with the app program within the 8-week intervention phase, however they did not display additional content, but rather served as a repetition of the contents from level 1–6 (i.e., the core program).

Criteria for advancing to the next level are (i) completing every exercise at least once, (ii) completing a sleep diary at least five times per level (iii) practicing audio relaxation techniques at least seven times per level. The design of the dCBT-I program ensures that a commitment of merely 10–15 min daily is sufficient in order to progress and thus facilitating seamless integration into daily routines. In this 8-week study, participants were advised to complete a minimum of the initial six levels of the app, strategically designed to encapsulate key features of CBT-I.

The distinguishing feature of the app is its dual sleep monitoring approach: subjectively through the daily sleep diary and objectively through a heart rate sensor worn daily during the night over the course of therapy. This dual sleep monitoring is integrated with dCBT-I interventions on a daily basis. Additionally, users receive daily feedback and individualized sleep recommendations based on their objectively measured sleep.

### Objective sleep measurement – heart rate sensor data

Continuous objective sleep monitoring was conducted by the app with the Polar® H10 heart rate sensor (Polar Electro; Kempele, Finland), which was worn around the chest at night and captured heart rate and inter heartbeat intervals (IBIs) in very high quality and on par with traditional two-channel electrocardiograms (Schaffarczyk et al., 2022). The gathered data were analysed by an AI-based algorithm, more specifically, a deep learning network for accurate 4-class sleep-staging (Wake, Light [N1+N2], Deep [N3], REM) based on heart rate variability (HRV) data, which has been validated against PSG (Topalidis et al., 2023a, 2023b). This allows for daily acquisition of “classical” sleep parameters such as total sleep time (TST), sleep efficiency (SE), wake after sleep onset (WASO), sleep onset latency (SOL), as well as information on the four sleep stages (duration, percentage, latency). Participants bought the sensor themselves for a reduced price (∼ 60€), which they could keep after the end of the study and were advised to wear the sensor and thus perform objective sleep measurement at least four times a week to ensure meaningful data collection.

### Subjective sleep measurement – sleep diary data

Upon awaking and retrieving the sensor data in the app in the morning, individuals were seamlessly directed to document their sleep in a short sleep diary, consisting of 5 questions assessing subjective sleep quality, subjective SOL as well as the subjective number of nocturnal awakenings (NOA) and subjective WASO. After filling in the sleep diary participants were provided with a comprehensive summary of the gathered information, including both subjective and objective sleep metrics, as well as a hypnogram depicting the night’s sleep patterns and a weekly synopsis. To contextualize their sleep data, they were also presented with normative sleep metrics for healthy counterparts matching their sex and age group.

### Questionnaires

For all three main testing points, i.e., pre-intervention (T1), post-intervention (T2) as well as the follow-up assessment (T3), the following questionnaires were completed: Pittsburgh Sleep Quality Index (PSQI; (Buysse et al., 1989)) for measuring subjective sleep quality; Insomnia Severity Index (ISI; (Dieck et al., 2018)) for assessing the severity of insomnia complaints; the Dysfunctional Beliefs and Attitudes about Sleep Scale-16 (DBAS-16; (Weingartz & Pillmann, 2009)) to evaluate misconceptions related to sleep, insomnia, and medication reliance; the Brief Symptom Inventory (BSI; (Franke, 2000)) for measuring overall psychological distress (GSI) as well as depression and anxiety (using the according subscales of the BSI), and the short version of the WHO Quality of Life Questionnaire (WHOQOL-BREF; (World Health Organization, 1996)) for evaluating physical health, psychological health, social interactions and environmental factors.

Additionally, at pre-intervention we assessed personality traits using the Big Five Inventory (BFI-10) (Rammstedt et al., 2014) and technical affinity using the Affinity for Technology Interaction (ATI) (Franke et al., 2019) a unidimensional self-report questionnaire assessing the individual's predisposition to interact with technology.

### Data analysis

Statistical analysis was performed in SPSS 29 (IBM Corp, 2021). Non-parametric tests - Friedman test and Wilcoxon test – were applied in order to not exclude extreme, but plausible values, and as we analyzed ordinally scaled variables (questionnaire data). The Wilcoxon tests effect size (r) was calculated by dividing the Z scores by the square root of the sample size; r’s between 0.10 and 0.29 were interpreted as small effects, r’s between 0.30 and 0.49 as medium effects, and r’s larger than 0.5 as large effect sizes (Cohen, 1988).

The significance threshold at p < .05 was applied. However, as multiple comparisons were conducted on the same sample, the post-hoc significance threshold was Bonferroni-corrected for the non-parametric tests to 0.017 for the pairwise three time point measurement comparisons within questionnaire analysis; for the pairwise comparisons of the six levels it was set to 0.003.

For analysis of sleep diary and sensor data we used the first 5 nights with sensor data per participant and meaned the nights for a proxy of the participant’s sleep at the beginning of the study, and the mean of the last 5 recorded nights of the intervention phase as a proxy for the participant’s sleep at the end of the intervention. We decided to mean over 5 nights in order to get a more realistic estimate on the individual’s sleep and to account for potential surprisingly good or poor nights, without having to exclude plausible nights.

For the level analysis, we included participants who reached at least level 6 (n = 64), which marks the minimum number of levels needed to complete the main contents of CBT-I.

## Results

### Questionnaire data

#### Insomnia severity (ISI) and subjective sleep quality (PSQI)

For insomnia severity, Friedman tests with Time (T1, T2, T3) as repeated measure indicated a significant decrease in the severity of insomnia across the three measurement points (χ2(2) = 57.80, p < .001) with (i) significant decreases from pre-intervention to post-intervention, (ii) from post-intervention to follow up assessment, and (iii) from pre-intervention to follow up assessment.

**Fig. 3:**
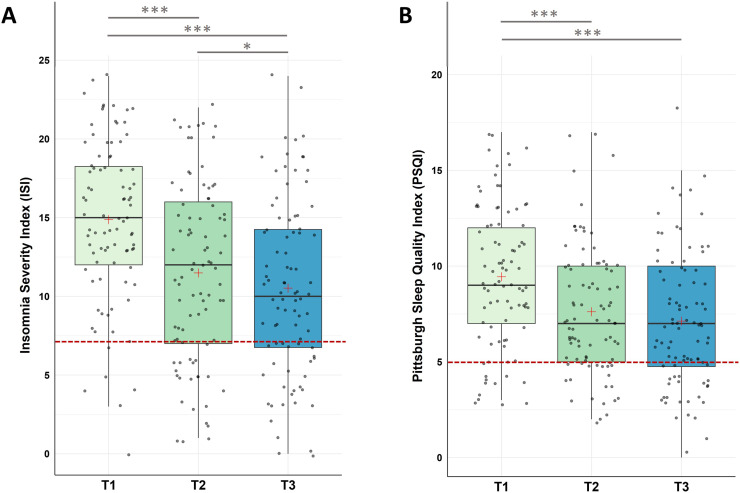
Changes in Insomnia Severity (ISI) and Subjective Sleep Quality (PSQI) Notes: A. Analysis revealed significant improvements in ISI scores during the intervention phase (T1-T2, p < 0.001), across the whole study (T1-T3, p < .001), as well as during the follow-up period (T2-T3, p = .007). B. Analysis revealed significant improvements in PSQI scores during the intervention phase (T1-T2, p < 0.001), across the whole study (T1-T3, p < .001), with no significant changes during the follow-up period (T2-T3, p = .041). Higher values represent a stronger impairment. The red line represents the Cut-Off value to normal/healthy sleep (ISI ≤ 7; PSQI ≤ 5). Horizontal lines represent the medians, boxes the interquartile range, with whiskers depicting the 1.5 interquartile range. The red cross corresponds to the mean. Abbreviations: T1: Start of the study/pre-intervention; T2: post-intervention; T3: follow-up assessment. Asterisks indicate significance (Bonferroni-corrected for multiple testing with 3 pairwise comparisons): *** p < .001, ** p < .003 * p < .017.

For subjective sleep quality, Friedman test detected a significant decrease in the Pittsburgh Sleep Quality Index (PSQI) across the three time points (χ2(2) = 31.010, p < .001). Post-hoc analysis found significant decreases (i) from pre-intervention to post-intervention (T1-T2), (ii) from pre-intervention to follow-up (T1-T3), however, (iii) not from post-intervention to follow-up (T2-T3; see Fig. 3 and Table 1 for descriptive values).

**Table 1:** Summary of Questionnaire results (N = 88).

Questionnaire	T1 (M±SD)	T2 (M±SD)	T3 (M±SD)	Friedman test	Wilcoxon test (post hoc)		
					T1-T2	T1-T3	T2-T3
ISI	14.88 ± 5.09	11.49 ± 5.66	10.52 ± 5.64	χ2(2) = 57.80, p<.001 ***	Z=−5.57, p<.001, r=−0.59 ***	Z=−6.32, p<.001, r=−0.67 ***	Z=−2.72, p=.007, r=−0.29 *
PSQI	9.44 ± 3.67	7.61 ± 3.34	7.12 ± 3.55	χ2(2) = 31.01, p<.001 ***	Z=−4.82, p<.001, r=−0.51 ***	Z=−5.54, p<.001, r=−0.59 ***	Z=−2.05, p=.041, r=−0.22
DBAS-16	4.77 ± 1.81	4.23 ± 1.91	4.08 ± 2.06	χ2(2) = 20.10, p<.001 ***	Z=−4.05, p<.001, r=−0.43 ***	Z=−4.50, p<.001 r=−0.48 ***	Z=−0.84, p=.401, r=−0.09
BSI – GSI	0.47 ± 0.42	0.39 ± 0.37	0.38 ± 0.39	χ2(2) = 17.10 p<.001 ***	Z=−3.01, p=.003, r=−0.32 *	Z=−4.04, p<.001, r=−0.43 ***	Z=−0.39, p=.697, r=−0.04
BSI – Depression	0.47 ± 0.57	0.36 ± 0.55	0.38 ± 0.59	χ2(2) = 10.59 p = .005 **	Z=−2.56, p=.010, r=−0.27 *	Z=−2.29, p=.022, r=−0.24	Z=−0.48, p=.630, r=−0.05
BSI – Anxiety	0.54 ± 0.54	0.41 ± 0.43	0.38 ± 0.46	χ2(2) = 8.40 p = .015 *	Z=−2.21, p=.027, r=−0.24	Z=−3.01, p=.003, r=−0.32 *	Z=−1.28, p=.201, r=−0.14
WHOQOL – Physical	69.47 ± 12.97	73.86 ± 14.67	74.10 ± 14.12	χ2(2) = 14.60 p<.001 ***	Z=−3.46, p<.001, r=−0.37 ***	Z=−3.24, p=.001, r=−0.35 **	Z=−0.35, p=.725, r=−0.04
WHOQOL – Psychological	67.67 ± 16.30	69.88 ± 16.41	72.49 ± 16.50	χ2(2) = 13.00 p = .002 **	Z=−1.56, p=.057, r=−0.17	Z=−3.66, p<.001, r=−0.39 ***	Z=−2.62, p=.009, r=−0.28 *
WHOQOL – Social	63.49 ± 21.45	68.99 ± 20.17	69.19 ± 22.22	χ2(2) = 10.55 p = .005 **	Z=−2.81 p=.005, r=−0.30 *	Z=−3.07, p=.002, r=−0.33 **	Z=−0.36, p=.716, r=−0.04
WHOQOL – Environment	84.24 ± 12.08	85.00 ± 11.00	85.44 ± 11.96	χ2(2) = 1.43 p = .490	Z=−0.41, p=.682, r=−0.04	Z=−1.05, p=.294, r=−0.11	Z=−0.76, p=.449, r=−0.08

Notes: Abbreviations: BSI-Anxiety: Brief Symptom Inventory – subscale anxiety; BSI-Depression: Brief Symptom Inventory – subscale depression; BSI – GSI: Brief Symptom Inventory – global score; DBAS-16: Dysfunctional beliefs and Attitudes About Sleep; ISI: Insomnia Severity Index; PSQI: Pittsburgh Sleep Quality Index; T1: pre-intervention; T2: post-intervention; T3: follow-up assessment; WHOQOL – Physical: WHO Quality of Life – physical quality of life; WHOQOL – Psychological: WHO Quality of Life – psychological quality of life; WHOQOL – Social: WHO Quality of Life – social relationships/support

Asterisks indicate significance: For Friedman tests: *** p < .001, ** p < .01, * p < .05; for post-hoc Bonferroni-adapted Wilcoxon tests as pairwise comparisons (p<.05 divided by the number of multiple comparisons (3 per questionnaire)): *** p < .001, ** p < .003 * p < .017.

#### Dysfunctional beliefs about sleep (DBAS-16) and psychological strain (BSI)

For dysfunctional beliefs and attitudes about sleep, the non-parametric Friedman Test along the repeated measures factor Time (T1, T2, T3) detected a significant decrease in DBAS-16 along the three time measurement points (χ2(2) = 20.10, p < .001) with significant decreases (i) from pre-intervention to post-intervention (T1-T2), and (ii) from pre-intervention to follow-up assessment (T1-T3). No significant changes were found (iii) for the follow-up period (T2-T3).

For psychological strain, non-parametric Friedman Tests with the repeated measures factor Time (T1, T2, T3) showed significant changes in participants general psychological distress (GSI) across the designated time points (χ2(2) = 17.10, p < .001), in depression (χ2(2) = 10.60, p = .005), and in anxiety levels (χ2(2) = 8.40, p = .015).

Post-hoc pairwise comparisons found a significant decrease in the BSI global score (GSI) between (i) pre-intervention and post-intervention (T1-T2), and (ii) pre-intervention and the end of the follow-up period (T1-T3); no significant changes were found for the follow-up period (T2-T3).

In the BSI subscale depression significant decreases were found between (i) pre-intervention and post-intervention (T1-T2); no significant changes were found for (ii) pre-intervention and the end of the follow up period (T1-T3) and the follow-up period (T2-T3).

**Fig. 4:**
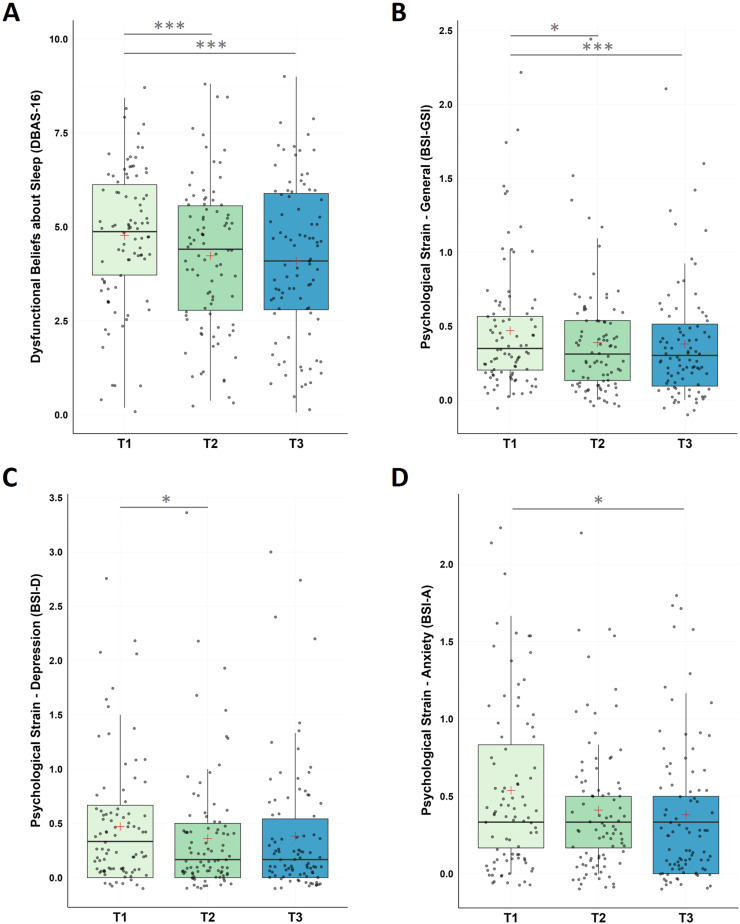
Changes in Dysfunctional Beliefs about Sleep (DBAS-16) and Psychological Strain (BSI) Notes: A. Analysis revealed significant improvements in DBAS-16 scores during the intervention phase (T1-T2, p < 0.001), across the whole study (T1-T3, p < .001), with no significant changes during the follow-up period (T2-T3, p = .401). B. Analysis revealed significant improvements in BSI-GSI scores during the intervention phase (T1-T2, p = 0.003), across the whole study (T1-T3, p < .001), with no significant changes during the follow-up period (T2-T3, p = .697). C. BSI-Depression scores significantly improved within the intervention phase (T1-T2, p = .010), with no significant changes from T1-T3 (p = .022) and from T2-T3 (p = .630). D. For BSI-Anxiety, participants improved across the study (T1-T3, p = .003), with no significant changes between T1-T2 (p = .027) and T2-T3 (p = .201). Higher values represent a stronger impairment. Horizontal lines represent the medians, boxes the interquartile range, with whiskers depicting the 1.5 interquartile range. The red cross corresponds to the mean. Abbreviations: T1: Start of the study/pre-intervention; T2: post-intervention; T3: follow-up assessment. Asterisks indicate significance (Bonferroni-corrected for multiple testing with 3 pairwise comparisons): *** p < .001, ** p < .003 * p < .017.

In the BSI subscale anxiety significant decreases were found between (i) pre-intervention and the end of the follow-up period (T1-T3), however, no changes were found (ii) from pre- to post-intervention (T1-T3) and (iii) during the follow-up period (T2-T3; see Fig. 4 and Table 1).

#### Quality of life (WHOQOL)

The non-parametric Friedman Test detected significant changes in physical health (χ2(2) = 14.60, p < .001), with significant increase from (i) pre-intervention to post-intervention, (ii) pre-intervention to follow-up assessment, however, no changes were found for the follow-up period.

Moreover, we found significant changes in psychological health (χ2(2) = 13.00, p = .002) with significant increase from (i) pre-intervention to follow-up assessment (T1-T3), and between (ii) post-intervention to follow-up assessment (T2-T3); no significant changes were found between (iii) pre-intervention and post-intervention (T1-T2).

The social relationships domain revealed significant changes (χ2(2) = 10.55, p = .005) along the three time measurement points with significant increase from (i) pre-intervention and post-intervention (T1-T2), and from (ii) pre-intervention and at the end of the follow-up period (T1-T3), however, not during the follow-up period (T2-T3).

**Fig. 5:**
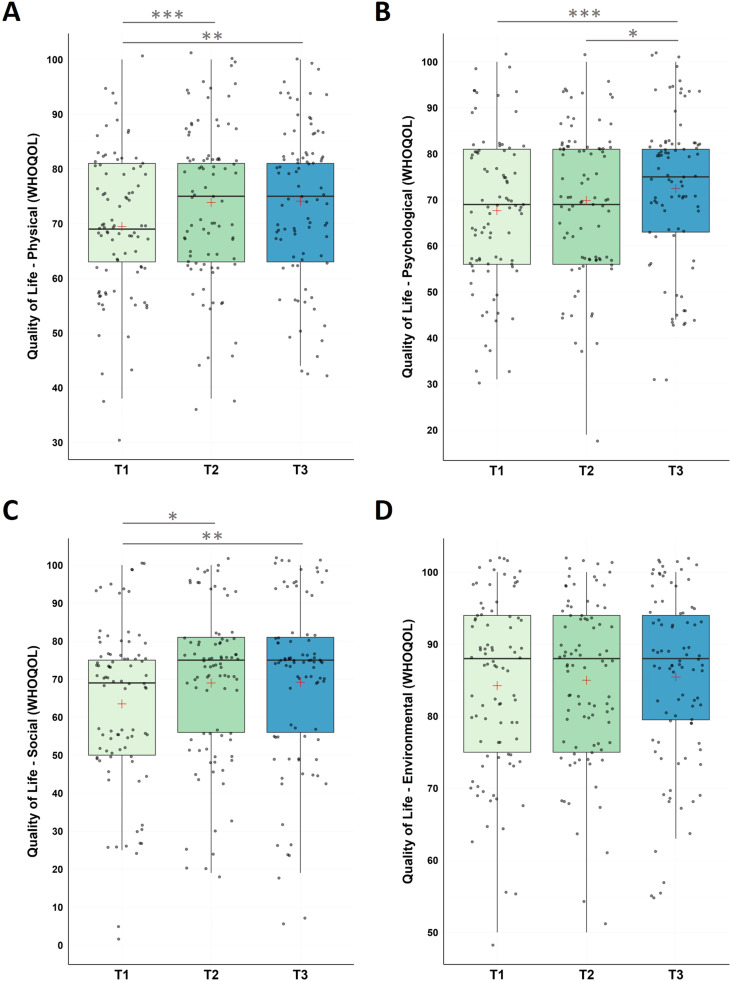
Changes in Quality of Life (WHOQOL) Notes: Analysis revealed significant improvements in WHOQOL (physical QOL) scores during the intervention phase (T1-T2, p < 0.001), across the whole study (T1-T3, p = .001), with no significant changes during the follow-up period (T2-T3, p = .725). WHOQOL (psychological QOL) scores significantly improved across the study (T1-T3, p < .001) and the follow-up period (T2-T3, p = .009), with no significant changes from T1-T2 (p = .057). For WHOQOL (social QOL), participants improved during the intervention phase (T1-T2, p = 0.005), across the whole study (T1-T3, p = .002), with no significant changes during the follow-up period (T2-T3, p = .716). WHOQOL (environmental QOL) did not change significantly across neither of the testing points (p’s >0.294). Higher values represent a stronger impairment. Horizontal lines represent the medians, boxes the interquartile range, with whiskers depicting the 1.5 interquartile range. The red cross corresponds to the mean. Abbreviations: QOL: Quality of Life; T1: Start of the study/pre-intervention; T2: post-intervention; T3: follow-up assessment. Asterisks indicate significance (Bonferroni-corrected for multiple testing with 3 pairwise comparisons): *** p < .001, ** p < .003 * p < .017.

For environmental factors, we did not find significant changes across the study (χ2(2) = 1.43, p = .490; see Fig. 5 and Table 1 for descriptive statistics).

### Pre- to post-intervention (first 5 vs. last 5 recorded nights)

#### Subjective daily sleep diary data

The sleep diary data analysis revealed improvements, i.e., significant increases in (i) subjective sleep quality and (ii) subjective sleep efficiency (SE), and significant decreases in (iii) subjective sleep onset latency (SOL), (iv) subjective number of awakenings (NOA) and (v) subjective wake after sleep onset (WASO). In addition, we found a reduction in subjective time in bed (TIB) and a statistical trend towards a reduction in subjective total sleep time (TST); the Sleep Score (combined value including sleep duration, sleep regularity and subjective sleep quality, scores between 1 and 10, while higher scores represent more favourable values), however, did not change significantly (see Table 2 for descriptive values and statistics).

**Table 2:** Summary of subjective sleep diary results (N = 85).

Sleep Parameter	Beginning (T1) (M±SD)	End (T2) (M±SD)	Wilcoxon-Test
Subjective Sleep Quality	5.60 ± 1.24	6.62 ± 1.37	Z = −5.95, p < .001, r = −0.65 ***
Subjective SE ( %)	84.28 ± 22.63	86.51 ± 8.78	Z = −4.45, p < .001, r = −0.48 ***
Subjective SOL (mins)	28.86 ± 22.37	21.23 ± 17.84	Z = −4.54, p < .001, r = −0.49 ***
Subjective NOA	2.77 ± 1.64	2.27 ± 1.62	Z = −3.92, p < .001, r = −0.43 ***
Subjective WASO (mins)	41.18 ± 33.61	29.04 ± 27.66	Z = −4.17, p < .001, r = −0.45 ***
Subjective TST (mins)	393.72 ± 64.60	406.26 ± 56.36	Z = −1.95, p = .051, r = −0.21 +
Subjective TIB (mins)	478.72 ± 52.64	468.83 ± 49.23	Z = −2.05, p = .041, r = −0.22 *
Sleep Score	6.31 ± 0.90	6.21 ± 1.15	Z = −0.58, p = .562, r = −0.06

Notes: Beginning = First 5 recorded nights of the intervention phase; End = Last 5 recorded nights of the intervention phase. Sleep Quality: Scores between 1 (very poor) and 10 (very good). Sleep Score: Combined value including sleep duration, sleep regularity and subjective sleep quality; Scores between 1 (very poor) and 10 (very good. Abbreviations: NOA: Number of awakenings; SE: Sleep Efficiency; SOL: Sleep Onset Latency; TIB: Time in Bed; TST: Total Sleep Time; T1: pre-intervention; T2: post-intervention; WASO: Wake After Sleep Onset. Asterisks indicate significance: *** p < .001, ** p < .01, * p < .05.

+ p < 0.1.

#### Objective daily sensor data

The analysis of objective data revealed statistical trends for a decrease in TST (M ± SD_Beginning_ = 405.55 ± 50.34; M ± SD_End_ = 395.58 ± 55.41; Z = −1.73, p = .083, r = −0.19) and Light Sleep in minutes (M ± SD_Beginning_ = 246.27 ± 36.19; M ± SD_End_ = 239.18 ± 37.42; Z = −1.70, p = .089, r = −0.18). Other objective sleep parameters, including SE, SOL, NOA, WASO, Wake After Final Awakening (WAFA), TIB, Light Sleep in % of TST, Deep Sleep in minutes and % of TST as well as REM Sleep in minutes and % of TST did not change significantly (see Supplementary Table S1 for descriptive values and statistics).

### Level by level analysis (continuous sleep monitoring during intervention)

Continuous sleep monitoring over the course of the 8-week intervention phase allowed for a closer look into changes in sleep patterns throughout the dCBT-I program (i.e., the 6 levels). As the program is structured in levels, we meaned the sleep values within one level per person to get an estimate of the participant’s sleep in the according level and compared the levels with each other.

#### Subjective data - sleep efficiency and wake after sleep onset

**Fig. 6:**
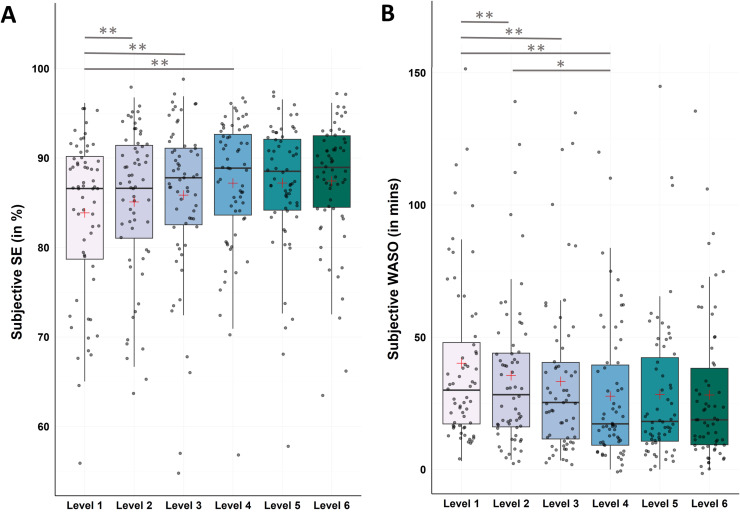
Changes in subjective sleep efficiency (SE) and wake after sleep onset (WASO) Notes: N = 62. A. Analysis revealed significant improvements in subjective SE from level 1 to level 4 (p < .001), to level 5 (p < .001), and to level 6 (p < .001). B. Analysis revealed significant improvements in subjective WASO from level 1 to level 4 (p < .001), to level 5 (p < .001), and to level 6 (p < .001), as well as from level 2 to level 4 (p = .002). Horizontal lines represent the medians, boxes the interquartile range, with whiskers depicting the 1.5 interquartile range. The red cross corresponds to the mean. Abbreviations: WASO: Wake After Sleep Onset. Asterisks indicate significance (Bonferroni-corrected for multiple testing with 15 pairwise comparisons): ** p < .001 * p < .003.

For SE Friedman test revealed significant changes between levels (χ2(5) = 28.97, p < .001). Post-hoc Bonferroni-corrected Wilcoxon tests showed significant increases from level 1 to level 4 (M ± SD_Level1_ = 83.87 ± 8.70; M ± SD_Level4_ = 87.20 ± 7.36; Z = −4.28, p < .001, r = −0.54), to level 5 (M ± SD_Level5_ = 87.20 ± 7.01; Z = −3.50, p < .001, r = −0.44), and to level 6 (M ± SD_Level6_ = 87.44 ± 7.22; Z = −3.60, p < .001, r = −0.46; see Fig. 6A and Supplementary Table S2 for all descriptive values and post-hoc comparisons).

For WASO Friedman test revealed significant changes between levels (χ2(5) = 44.29, p < .001). Post-hoc Bonferroni-corrected Wilcoxon tests showed significant decreases from level 1 to level 4 (M ± SD_Level1_ = 40.19 ± 31.72; M ± SD_Level4_ = 27.73 ± 26.50; Z = −5.21, p < .001, r = −0.66), to level 5 (M ± SD_Level5_ = 28.38 ± 27.57; Z = −3.69, p < .001, r = −0.47), and to level 6 (M ± SD_Level6_ = 28.18 ± 28.25; Z = −4.46, p < .001, r = −0.57), as well as from level 2 to level 4 (M ± SD_Level2_ = 35.53 ± 29.18; Z = −3.13, p = .002, r = −0.40; see Fig. 6B and Supplementary Table S3 for all descriptive values and post-hoc comparisons).

#### Objective data – total sleep time

**Fig. 7:**
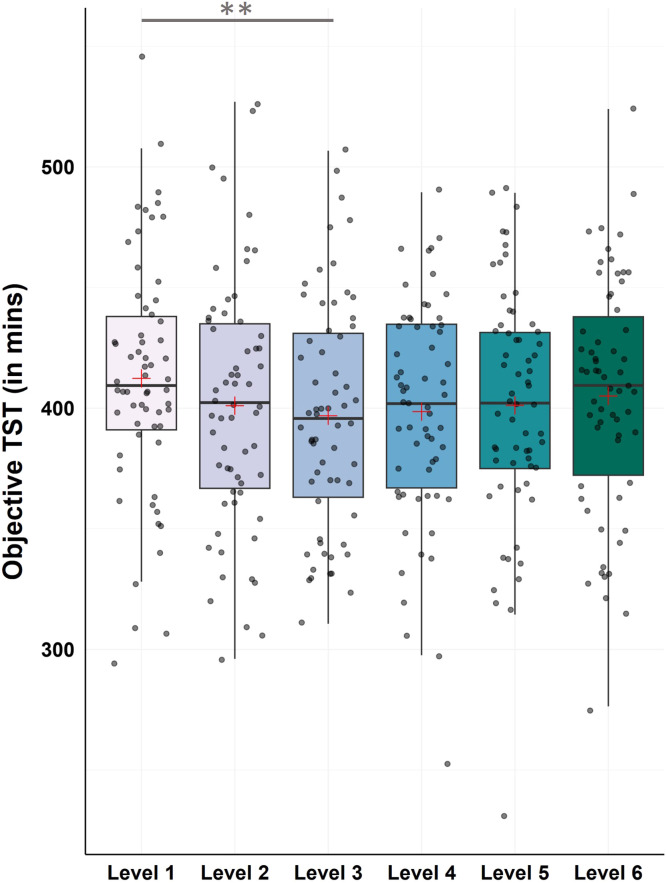
Changes in objective total sleep time (TST) Notes: N = 62. Analysis revealed significant improvements in objective TST from level 1 to level 3 (p < .001). Horizontal lines represent the medians, boxes the interquartile range, with whiskers depicting the 1.5 interquartile range. The red cross corresponds to the mean. Abbreviations: TST: Total Sleep Time. Asterisks indicate significance (Bonferroni-corrected for multiple testing with 15 pairwise comparisons): ** p < .001 * p < .003.

For TST the Friedman test revealed a statistical trend towards changes between levels (χ2(5) = 11.05, p = .050). Post-hoc Bonferroni-corrected Wilcoxon tests showed a significant decrease from level 1 to level 3, which marks the introduction of the CBT-I sleep restriction intervention (M ± SD_Level1_ = 412.35 ± 49.18; M ± SD_Level3_ = 396.84 ± 47.57; Z = −3.40, p < .001, r = −0.43; see Fig. 7 and Supplementary Table S4 for all descriptive values and remaining post-hoc comparisons).

## Discussion

We investigated the efficacy of a smartphone app program combining unguided dCBT-I with continuous subjective and objective sleep measurement in the field, i.e., in a more naturalistic rather than a standardized clinical setting. We hereby analyzed questionnaire data, subjective sleep-diary derived, as well as objective HR sensor derived sleep parameters across the 8-week intervention period.

The dCBT-I intervention showed to be effective in reducing insomnia symptoms, as the sample on average changed from moderate insomnia severity to subthreshold insomnia over the course of the intervention. Furthermore, insomnia prevalence decreased from 92 % at pre-intervention to 70,5 % post-intervention, with only 1.1 % (n = 1) experiencing severe clinical insomnia, 31.8 % (n = 28) experiencing moderate insomnia, 37.5 % (n = 33) experiencing subthreshold insomnia, and 29.5 % (n = 26) not having clinically significant sleep issues after the intervention. These effects were stable at the end of the follow-up, with a further decrease to 67 %. A 6-point reduction in ISI scores is considered a minimally important difference (MID) for a community sample to represent a clinically meaningful improvement in individuals with primary insomnia (Yang et al., 2009). In the present study, out of the 88 participants, 27.3 % (n = 24) experienced clinically meaningful improvement between pre-intervention and post-intervention, and 40.9 % (n = 36) reported improvement between pre-intervention and at the follow-up. Overall, 44.31 % were identified as responders during the study.

Subjective sleep quality, as measured with the PSQI, significantly improved post-intervention, although it remained in the category of poor sleep quality, indicating an effect of the intervention on improving sleep quality yet suggesting that longer treatment might lead to further benefits. Additionally, we found a sustained reduction in dysfunctional beliefs and attitudes about sleep, and participants reported lower levels of psychological distress. The intervention also had a positive effect on the participants’ well-being, as indicated by reduced levels of depression and anxiety, and improvements in different aspects of quality of life, including perceived physical health, psychological health and social relationships.

Regarding continuous sleep measurement during the intervention, as expected, subjective sleep diary-derived data showed significant or tendentious improvements on almost every parameter, indicating that participants perceived their sleep as better and overall were more satisfied with their sleep at the end of the study. Only the Sleep Score, a combined value of three parameters, did not change significantly. This may be due the calculation of the Sleep Score, as it does not only comprise subjective sleep quality, but also sleep regularity and sleep duration, which includes objective, sensor-derived values. As our analyses have shown, objective changes were substantially more difficult to detect when compared to subjective changes, which is a common phenomenon described in the literature (e.g., Mitchell et al., 2019). Apart from the fact, that subjective improvements are quicker to be found and are usually more profound, it is also important to keep the baseline values of objective sleep parameters in mind in order to understand whether changes and improvements were likely to happen in the first place. Following the desired treatment outcomes by Schutte-Rodin and colleagues, SOL and WASO should be under 30 min, TST over 6 h and SE at least between 80 and 85 % after treatment (Schutte-Rodin et al., 2008). When comparing these values with objective baseline characteristics of our sample (see Supplementary Table S1), it shows that our sample was mostly in a healthy range at pre-intervention already (SE = 86 %, SOL = 16 min, TST = 6.75 h), meaning that substantial improvements were unlikely to happen. Only baseline WASO exceeded those values with 52 mins, which is comparable with the average objective WASO of 58 min in insomnia patients as investigated in a meta-analysis (Baglioni et al., 2014).

Overall, we found large effect sizes for insomnia severity, sleep quality and dysfunctional beliefs about sleep, medium effect sizes for symptoms of anxiety, SE, SOL, NOA and WASO, as well as small effect sizes for symptoms of depression and changes in subjective TST. Those findings are mostly in line with other studies investigating digital CBT-I programs (e.g., large effect sizes for insomnia severity as well as preoccupation about sleep and medium effect size for anxiety in Lorenz et al. (2019); large effect sizes for SE and WASO, medium effect sizes for SOL, TST and sleep quality in Espie et al. (2012), making the results comparable to other well-established digital CBT-I programs.

The inclusion of continuous sleep measurement in a digital therapy program fosters the understanding of changes in sleep patterns over the course of an intervention, that might be missed when only investigating the changes from beginning to the end of an intervention. While the latter is important to evaluate the efficacy of a therapy program and draw conclusions for revenant treatment outcomes for patients, a lot of information about events within the therapy phase is potentially lost. In the present study, we therefore also investigated the sleep pattern of participants on a therapy level-by-therapy level basis, which allowed for a stable estimate of a person’s change within one level. Following this approach, we found that the biggest changes in subjective SE and WASO occurred within the first three to four levels of the therapy program and stayed relatively stable over the rest of the intervention phase. Similarly, our results showed a significant drop in objective TST from level 1 to level 3. This specific result provides insight into the content of the therapy program: The drop in TST in level 3 corresponds to the treatment content in this level, i.e., the introduction of the sleep restriction (SR) intervention. By observing the drop in TST we can conclude that the sample on average actually followed the SR instructions, despite the program being unguided.

It is, however, important to be noted, that the comparison of pre- to post-intervention (i.e., the first 5 and last 5 recorded nights), does not necessarily reflect the same effects as the comparison of level 1 with level 6, as participants have reached different progress during the 8-week intervention phase: 74 % of the sample reached (at least) level 6, which marks the desired level for participants to learn about the most important CBT-I contents, and 56 % completed level 6 (i.e., completed the full program), meaning that at “post-treatment” some participants were still in level 1–5 (see Supplementary Table S5 for an overview over the therapy program progress). This could explain potential carry-over effects of participants who started the SR later in the intervention phase rather than during the middle as intended (in level 3). This could also explain the statistical trend towards a reduction of objective TST from pre- to post intervention, as some participants finished their intervention phase on level 3 or 4, so only shortly after the introduction of SR.

The inclusion of continuous sleep measurement represents a necessity in order to fully capture and understand a patient’s sleep pattern and changes over the course of the therapy. While various digital applications for insomnia therapy include a sleep diary, which corresponds to subjective continuous sleep measurement, we argue, that the combination with an objective continuous measure, as used in the NUKKUAA/sleep² program, does not only add a valuable addition to collecting more informative data, but is also necessary in order to fully understand a patient’s sleep pattern and changes thereof. For example, only by including both subjective and objective measurement, it is possible to investigate the discrepancy of subjective and objective sleep parameters, a common phenomenon in insomnia disorder, and the improvement thereof during therapy (Hinterberger et al., 2023). Even in our data we find tendencies of this discrepancy, as we found a tendentious decrease in objective TST from pre- to post-intervention, yet a tendentious increase in self-reported TST during the same time. To summarize, the inclusion of validated HRV-based continuous objective sleep measurement provides a low-cost and effective alternative to resource-intensive PSG, when assessing changes in objective sleep parameters. In this study, we acquired a total of 3460 nights with the HR-sensor across all participants and all levels, with participants using the HR-sensor on 6.4 days per level within the core program (level 1–6) on average. This allowed for a stable objective estimate of a person’s sleep and therefore reliable interpretation of sleep changes during intervention.

It is to be noted, that sleep measurement using wearables has already been incorporated into some digital CBT-I studies, however, it is crucial to distinguish how these devices are used: solely for objective sleep assessment, as part of an active feedback loop, or in a continuous monitoring framework. Moreover, many studies differ in whether they report data from ongoing wearable recordings or focus primarily on pre- to post-intervention outcomes. For instance, Kang et al. (2017) tested a mobile CBT-I app synchronizable with a wearable device, but it remained unclear whether participants received individualized feedback or if the wearable functioned mainly as an objective measurement tool. Luik et al. (2018) compared users of a large-scale digital CBT-I program who did or did not connect a wearable; both groups improved similarly, though wearable users showed higher engagement. In contrast, Ito-Masui et al. (2023) integrated wearable-based sleep data into an app for shift workers, finding improvements in sleep duration and subjective sleep quality. These variations highlight that the role and reporting of wearables in digital CBT-I remain highly heterogeneous and is therefore yet to be systemically investigated.

For an adequate interpretation of our results, it is important to keep in mind that our participants can be considered a sleep-disturbed, yet not a clinical sample as only 52 % had moderate to severe clinically relevant insomnia (ISI), and only 38,6 % scored beyond 10 on the PSQI and therefore qualified as chronic poor sleeper pre-intervention.

In unguided programs and study designs as in the present one, technical difficulties or (moderate) data loss due to app user mistakes as accidently mispositioning the HR sensor or starting or ending the nightly sleep measurement too early or too late, as well as occasional app connection issues, are inevitable, yet was rather minimal in the present study. Our app-data showed that participants indeed spend most days in the first level of therapy (11 ± 7.14, see Supplementary Table S5), most probably due to “figuring” out how to best work with the app and finding a suitable routine to incorporate the therapy nuggets in their daily life. After reaching the second level, the average days spent per level were around the expected amount of ± 7 days.

Some additional points regarding the sample are important to mention: Despite the representative gender distribution with more than half of participants being women, and half of the sample taking medication (ABDA, 2025), nearly 64 % of participants had a university degree. Therefore, the sample is to be considered highly educated, raising questions regarding the generalizability of the results and effects found in the present study. Furthermore, as sleep disturbances were not defined as a requirement in order to enter the study protocol, yet sample characteristics showed poor subjective sleep quality according to the PSQI in 83 % of participants and self-reported insomnia complaints of at least sub-threshold severity according to the ISI at the beginning of the study, a potential self-selection bias cannot be ruled out. It can be assumed that this sample was therefore potentially more motivated to participate and/or complete the study protocol, as compared to a randomly selected sample form the community.

However, the question is whether this self-selection bias is a limiting factor here: In considering the potential users of digital CBT-I programmes, as investigated in this study, it can be hypothesised that the programmes will be primarily sought after and utilised by individuals experiencing sleep disturbances, specifically insomnia complaints. Therefore, this bias could in fact reflect the characteristics of the real-world target population. Rather than representing a methodological limitation, it may thus be interpreted as a learning opportunity, providing valuable insights into the user profiles, engagement patterns, and optimisation potential of digital CBT-I interventions within naturalistic settings.

As mentioned earlier we did not include a control group due to reasons of compliance in the patients’ homes and much more naturalistic setting. Interpreting (subjective) results without a control condition raises the suspicion of potential placebo effects or improvement due to expectancy bias of the participants. However, the results follow an earlier randomized controlled trial investigating this app program’s effects on sleep (Hinterberger et al., 2023), as well as an additional study also investigating treatment effects on psychological distress and quality of life (Hinterberger et al., 2025). We therefore cautiously conclude that by combining digital CBT-I with both subjective and objective continuous sleep measurement the NUKKUAA/sleep² app program shows notable effects on various parameters and in different study settings and most importantly allows insights into specific intervention effects over the course of treatment.

## Declaration of competing interest

MS is Co-Founder and CSO of NUKKUAA®/sleep².

AH and E-SE received an honorarium for data collection from the NUKKUAA GmbH.

AS and PT declare no conflicts of interest.

## Appendix Supplementary materials

Image, application 1
