# Supplementary material for: Continuous sleep tracking in digital CBT-I: Efficacy and insights from a naturalistic-environment study
Source: Int J Clin Health Psychol. 2025 Nov 6;25(4):100646. doi: 10.1016/j.ijchp.2025.100646 (PMC12766055; doi:10.1016/j.ijchp.2025.100646)
Supplement: Supplementary file 1 [file mmc1.docx]

**Continuous Sleep Tracking in Digital CBT-I: Efficacy and Insights from a Naturalistic-Environment Study**

Table of Contents

[Supplementary Table S1 – *Summary of Objective Sensor Results (N = 85)* 3](#_Toc212740039)

[Supplementary Table S2 – *Summary of Subjective SE Results including pairwise comparisons (N = 62)* 4](#_Toc212740040)

[Supplementary Table S3 – *Summary of Subjective WASO Results including pairwise comparisons (N = 62)* 5](#_Toc212740041)

[Supplementary Table S4 – *Summary of Objective TST Results including pairwise comparisons (N = 62)* 6](#_Toc212740042)

[Supplementary Table S5 – *Overview of participant flow and number of HR-sensor nights within therapy levels* 7](#_Toc212740043)

# Supplementary Table S1 – *Summary of Objective Sensor Results (N = 85)*

| Sleep Parameter | Beginning (T1)  (M±SD) | End (T2)  (M±SD) | Wilcoxon-Test |
| --- | --- | --- | --- |
|  |  |  |  |
| *Objective SE (%)* | 85.59  ± 6.93 | 84.75  ± 9.29 | Z = -0.35, p = .727, r = -.04 |
| *Objective SOL (mins)* | 16.42  ± 16.06 | 18.50  ± 19.77 | Z = -1.12, p = .263, r = -.12 |
| *Objective NOA* | 22.73  ± 7.16 | 21.83  ± 7.20 | Z = -1.59, p = .113, r = -.17 |
| *Objective WASO (mins)* | 52.42  ± 25.10 | 53.76  ± 34.69 | Z = -0.36, p = .718, r = -.04 |
| *Objective WAFA (mins)* | 10.76  ± 12.18 | 10.64  ± 11.45 | Z = -1.10, p = .272, r = -.12 |
| *Objective TST (mins)* | 405.55  ± 50.34 | 395.58  ± 55.41 | Z = -1.73, p = .083, r = -.19 ^+^ |
| *Objective TIB (mins)* | 474.39  ± 51.25 | 467.83  ± 49.52 | Z = -1.55, p = .121, r = -.17 |
| *Light Sleep (mins)* | 246.27  ± 36.19 | 239.18  ± 37.42 | Z = -1.70, p = .089, r = -.18 ^+^ |
| *Light Sleep (%)* | 61.08  ± 7.13 | 60.79  ± 6.74 | Z = -0.87, p = .384, r = -.09 |
| *Deep Sleep (mins)* | 63.82  ± 22.25 | 62.92  ± 22.83 | Z = -0.93, p = .355, r = -.10 |
| *Deep Sleep (%)* | 15.69  ± 5.22 | 15.84  ± 5.21 | Z = -0.92, p = .927, r = -.10 |
| *REM Sleep (mins)* | 95.47  ± 19.00 | 93.31  ± 19.33 | Z = -1.10, p = .272, r = -.12 |
| *REM Sleep (%)* | 23.23  ± 3.44 | 23.38  ± 3.23 | Z = -0.56, p = .574, r = -.06 |

*Notes: Beginning = First 5 recorded nights of the intervention phase, End = Last 5 recorded nights of the intervention phase. Light sleep includes stages N1 and N2. Abbreviations: NOA: Number of awakenings; REM: Rapid Eye Movement Sleep; SE: Sleep Efficiency; SOL: Sleep Onset Latency; TIB: Time in Bed; TST: Total Sleep Time; WAFA: Wake After Final Awakening (until getting out of bed); WASO: Wake After Sleep Onset. Asterisks indicate significance: *** p < .001, ** p < .01, * p < .05, ^+^ p <.1.*

# Supplementary Table S2 – *Summary of Subjective SE Results including pairwise comparisons (N = 62)*

| Level | Descriptives  (M±SD) | Level - post-hoc comparison | Wilcoxon Test |
| --- | --- | --- | --- |
|  |  |  |  |
| *1* | 83.87  ± 8.70 | ***1-2*** | Z = -1.38, p = .168, r = -.18 |
|  |  | ***1-3*** | Z = -2.65, p = .008, r = -.34 |
|  |  | ***1-4*** | Z = -4.28, p < .001, r = -.54 ** |
|  |  | ***1-5*** | Z = -3.50, p < .001, r = -.44 ** |
|  |  | ***1-6*** | Z = -3.60, p < .001, r = -.46 ** |
| *2* | 85.10  ± 8.23 | ***2-3*** | Z = -0.51, p = .611, r = -.06 |
|  |  | ***2-4*** | Z = -2.94, p = .003, r = -.37 |
|  |  | ***2-5*** | Z = -2.30, p = .021, r = -.29 |
|  |  | ***2-6*** | Z = -2.42, p = .016, r = -.31 |
| *3* | 85.86  ± 8.66 | ***3-4*** | Z = -1.83, p = .068, r = -.23 |
|  |  | ***3-5*** | Z = -1.34, p = .182, r = -.17 |
|  |  | ***3-6*** | Z = -1.70, p = .089, r = -.22 |
| *4* | 87.20  ± 7.36 | ***4-5*** | Z = -0.47, p = .641, r = -.06 |
|  |  | ***4-6*** | Z = -0.68, p = .494, r = -.09 |
| *5* | 87.20  ± 7.01 | ***5-6*** | Z = -0.56, p = .572, r = -.07 |
| *6* | 87.44  ± 7.22 | ***-*** | - |

*Notes: Abbreviations: M: Mean; SD: Standard deviation; SE; Sleep Efficiency.*

*Asterisks indicate significance: For post-hoc Bonferroni-adapted Wilcoxon tests as pairwise comparisons (p<.05 divided by the number of multiple comparisons (15 per sleep parameter)): ** p < .001 * p < .003.*

# Supplementary Table S3 – *Summary of Subjective WASO Results including pairwise comparisons (N = 62)*

| Level | Descriptives  (M±SD) | Level - post-hoc comparison | Wilcoxon Test |
| --- | --- | --- | --- |
|  |  |  |  |
| *1* | 40.19  ± 31.72 | ***1-2*** | Z = -1.66, p = .097, r = -.21 |
|  |  | ***1-3*** | Z = -2.88, p = .004, r = -.37 |
|  |  | ***1-4*** | Z = -5.21, p < .001, r = -.66 ** |
|  |  | ***1-5*** | Z = -3.69, p < .001, r = -.47 ** |
|  |  | ***1-6*** | Z = -4.46, p < .001, r = -.57 ** |
| *2* | 35.53  ± 29.18 | ***2-3*** | Z = -1.09, p = .275, r = -.14 |
|  |  | ***2-4*** | Z = -3.13, p = .002, r = -.40 * |
|  |  | ***2-5*** | Z = -2.42, p = .016, r = -.31 |
|  |  | ***2-6*** | Z = -2.95, p = .003, r = -.37 |
| *3* | 33.29  ± 30.19 | ***3-4*** | Z = -2.54, p = .011, r = -.32 |
|  |  | ***3-5*** | Z = -1.13, p = .219, r = -.14 |
|  |  | ***3-6*** | Z = -2.46, p = .014, r = -.31 |
| *4* | 27.73  ± 26.50 | ***4-5*** | Z = -0.67, p = .504, r = -.09 |
|  |  | ***4-6*** | Z = -0.27, p = .787, r = -.03 |
| *5* | 28.38  ± 27.57 | ***5-6*** | Z = -0.76, p = .449, r = -.10 |
| *6* | 28.18  ± 28.25 | ***-*** | - |

*Notes: Abbreviations: M: Mean; SD: Standard deviation; WASO: Wake After Sleep Onset.*

*Asterisks indicate significance: For post-hoc Bonferroni-adapted Wilcoxon tests as pairwise comparisons (p<.05 divided by the number of multiple comparisons (15 per sleep parameter)): ** p < .001 * p < .003.*

# Supplementary Table S4 – *Summary of Objective TST Results including pairwise comparisons (N = 62)*

| Level | Descriptives  (M±SD) | Level - post-hoc comparison | Wilcoxon Test |
| --- | --- | --- | --- |
|  |  |  |  |
| *1* | 412.35  ± 49.18 | ***1-2*** | Z = -2.45, p = .014, r = -.31 |
|  |  | ***1-3*** | Z = -3.40, p < .001, r = -.43 ** |
|  |  | ***1-4*** | Z = -2.18, p = .029, r = -.28 |
|  |  | ***1-5*** | Z = -2.00, p = .045, r = -.25 |
|  |  | ***1-6*** | Z = -1.50, p = .134, r = -.19 |
| *2* | 401.02  ± 51.63 | ***2-3*** | Z = -1.19, p = .236, r = -.15 |
|  |  | ***2-4*** | Z = -0.07, p = .947, r = -.01 |
|  |  | ***2-5*** | Z = -0.48, p = .629, r = -.06 |
|  |  | ***2-6*** | Z = -0.80, p = .422, r = -.10 |
| *3* | 396.84  ± 47.57 | ***3-4*** | Z = -0.85, p = .394, r = -.11 |
|  |  | ***3-5*** | Z = -1.34, p = .179, r = -.17 |
|  |  | ***3-6*** | Z = -1.65, p = .099, r = -.21 |
| *4* | 398.58  ± 45.93 | ***4-5*** | Z = -0.24, p = .814, r = -.03 |
|  |  | ***4-6*** | Z = -1.15, p = .249, r = -.15 |
| *5* | 401.14  ± 48.67 | ***5-6*** | Z = -1.04, p = .298, r = -.13 |
| *6* | 405.07  ± 48.82 | ***-*** | - |

*Notes: Abbreviations: M: Mean; SD: Standard deviation; TST; Total Sleep Time.*

*Asterisks indicate significance: For post-hoc Bonferroni-adapted Wilcoxon tests as pairwise comparisons (p<.05 divided by the number of multiple comparisons (15 per sleep parameter)): ** p < .001 * p < .003.*

# Supplementary Table S5 – *Overview of participant flow and number of HR-sensor nights within therapy levels*

| Level number | Number of participants who reached this level | % of participants who finished at this level | Calendar days spent on each level  (M ± SD) | Number of nights measured with HR-sensor in total | Average number of nights measured with HR-sensor per participant |
| --- | --- | --- | --- | --- | --- |
| 1 | 87 | 3.45% | 11 ± 7.14 | 720 | 8.28 |
| 2 | 84 | 3.45% | 9 ± 5.62 | 583 | 6.94 |
| 3 | 81 | 1.15% | 7 ± 2.57 | 495 | 6.11 |
| 4 | 80 | 9.20% | 7 ± 2.48 | 471 | 5.88 |
| 5 | 72 | 8.05% | 7 ± 2.14 | 424 | 5.89 |
| 6 | 65 | 18.39% | 7 ± 2.47 | 363 | 5.58 |
| 7 | 49 | 25.29 % | 6 ± 2.66 | 259 | 5.29 |
| 8 | 27 | 17.24% | 5 ± 2.55 | 107 | 3.96 |
| 9 | 12 | 11.49% | 3 ± 2.04 | 34 | 2.83 |
| 10 | 2 | 2.30% | 2 ± 1.41 | 3 | 1.50 |
